# Heat stress associated with aerosol PPE and its impact

**DOI:** 10.1093/occmed/kqac114

**Published:** 2022-10-25

**Authors:** N Chaudhari, P H Strutton, A J Wickham, A H McGregor, C J Mullington

**Affiliations:** MSk Lab, Imperial College London, London W12 0BZ, UK; MSk Lab, Imperial College London, London W12 0BZ, UK; Theatres and Anaesthesia, Imperial College Healthcare NHS Trust, London W2 1NY, UK; MSk Lab, Imperial College London, London W12 0BZ, UK; MSk Lab, Imperial College London, London W12 0BZ, UK; Theatres and Anaesthesia, Imperial College Healthcare NHS Trust, London W2 1NY, UK

## Abstract

**Background:**

Aerosol personal protective equipment (PPE) is subjectively reported to negatively impact healthcare workers’ performance and well-being, but this has not been assessed objectively.

**Aims:**

This randomized controlled crossover study aimed to quantify the heat stress associated with aerosol PPE and to investigate its impact upon mood, cognitive and motor function, and task performance.

**Methods:**

Sixteen healthy, young, lean participants (eight males) undertook an exercise protocol, which simulated the metabolic expenditure of hospital work: once wearing aerosol PPE (PPE visit) and once wearing standard surgical attire (control visit). Participants walked on a treadmill for 2 h followed by 30-min rest. Core temperature, heart rate, urine specific gravity, weight, grip strength, mood (Bond–Lader scale) and task performance (Intubation of a Manikin) were recorded. Values are between-visit mean (standard deviation) differences.

**Results:**

On the PPE visit core temperature (+0.2 (0.3)°C; *P* < 0.01), heart rate (+12 (13) bpm; *P* < 0.001), urine specific gravity (+0.003 (0.005); *P* < 0.05) and intubation task time (+50 (81) s; *P* < 0.01) were greater than on the control visit; and alertness (−14 (21) mm; *P* < 0.001), contentment (−14 (15) mm; *P* < 0.001) and grip strength (−4 (4) N; *P* < 0.01) were less.

**Conclusions:**

This study demonstrates that wearing aerosol PPE in a simulated hospital environment results in heat exhaustion and has a negative impact upon mood, motor function, and task performance. Whilst wearing PPE is important to prevent disease transmission, strategies should be developed to limit its impact upon healthcare workers’ performance and well-being.

## Teaser

During the COVID-19 pandemic, use of aerosol personal protective equipment (PPE) has increased. Whilst its use is necessary to protect healthcare workers from disease transmission, wearers have reported adverse effects on performance and well-being. This randomized controlled crossover study demonstrates that wearing aerosol PPE for 2 h in a simulated hospital environment results in heat exhaustion (core temperature >38°C, heart rate >100 bpm, excessive thirst and sweating) and impaired mood, muscle contraction and task performance.

**Key learning points**

**What is already known about this subject:**

- Aerosol personal protective equipment use has increased during the coronavirus disease-2019 pandemic.
- Subjectively aerosol personal protective equipment impairs healthcare workers’ performance as well as their physical and mental well-being, but objective measurements of its impact have not been conducted.
- Other forms of personal protective equipment are a source of heat stress, but it is not known how aerosol personal protective equipment affects body temperature.

**What this study adds:**

- Wearing aerosol personal protective equipment for 2 h results in hyperthermia (core temperature 38.1°C), tachycardia (heart rate 121 bpm) and dehydration (urine specific gravity 1.02).
- A decrement in mood and task performance is observed immediately after donning aerosol personal protective equipment.
- The heat stress associated with wearing aerosol personal protective equipment for 2 h has a negative impact on motor function and compounds the decrement in mood and task performance.

**What impact this may have on practice or policy:**

- Healthcare organizations should adopt strategies, such as mandatory breaks and buddy systems, to mitigate the risks associate with aerosol personal protective equipment use.
- Development and testing of heat-permeable personal protective equipment garments should be prioritized.

## Introduction

Medical personal protective equipment (PPE) is used to prevent transmission of infectious diseases. It is classified as ‘droplet’ or ‘aerosol’ [1]. Aerosol PPE is an enhanced form designed to protect healthcare workers when significant numbers of aerosol particles (<5 µm diameter) are generated, such as during endotracheal intubation. Prior to 2020, aerosol PPE was reserved for treating patients with a small range of aerosol-transmissible diseases, such as influenza. However, since the coronavirus disease-2019 (COVID-19) pandemic, its use has become more widespread, particularly in intensive care units, operating theatres and emergency departments.

Whilst the benefits of aerosol PPE use are clear, the negative consequences are less well-studied. One negative consequence is that it may inhibit cutaneous heat loss and thus induce heat stress. Heat stress is a condition in which heat production exceeds heat loss and, as a result, core temperature rises. Other forms of PPE are a heat stress, but it is not known how aerosol PPE affects body temperature [2,3]. If heat stress continues, it results in hyperthermia and dehydration, which negatively impacts mood, cognitive and motor function, and task performance [2–6]. Subjectively, aerosol PPE impairs performance in the workplace as well as physical and mental well-being, but objective evaluations of its impact on thermoregulation, cognition and motor function have not been conducted [7]. It is important to objectively understand the impact of wearing aerosol PPE because decrements in cognitive and motor function are associated with increased rates of medical error and repeated exposure to physically demanding conditions at work can lead to fatigue, burnout, and long-term physical and mental illness [8,9].

This randomized controlled crossover study aimed to quantify the heat stress associated with wearing aerosol PPE in a simulated hospital environment and to assess its effect upon mood, cognitive and motor function, and task performance. It was hypothesized that wearing aerosol PPE would result in hyperthermia and dehydration and that it would negatively impact mood, cognitive and motor function, and task performance.

## Methods

Ethical approval was obtained from the Imperial College Research Ethics Committee (20IC6445). Participants approached the study team after viewing poster advertisements at Imperial College London. After reading the participant information sheet, potential participants were given the opportunity to ask questions. Participants who fulfilled the inclusion and exclusion criteria and were happy to participate signed an informed consent form. Inclusion criteria: age 18–30 years; body mass index (BMI) 18–30 kg·m^−2^; and a negative COVID-19 infection screen. Exclusion criteria: pregnancy and any cardiovascular, gastrointestinal, neuromuscular or psychiatric disease.

Experiments were conducted in a human performance laboratory. Participants performed an exercise protocol on two occasions: once wearing aerosol PPE (PPE group) and once wearing standard surgical attire (control group). Aerosol PPE consisted of surgical scrubs, coverall, FFP3 respirator, visor and nitrile gloves; standard surgical attire consisted of surgical scrubs, type IIR face mask and surgical cap (Supplementary Figure 1, available as Supplementary data at *Occupational Medicine* Online) [1]. The visit order was randomized and, to negate learning effects, visits were scheduled approximately 1 week apart.

The exercise protocol consisted of 30-min acclimatization, 2-h walking on a treadmill (ZR11, Reebok, Boston, MA, USA), and 30-min rest. During the rest period participants consumed a 500-ml bottle of an electrolyte rehydration drink (Powerade, Coca-Cola, Atlanta, GA, USA). The treadmill speed was adjusted on the first visit during the first 5-min walking to elicit a heart rate that was 50% of the participant’s predicted maximum (maximum heart rate = 220 − age). This treadmill speed was maintained throughout the protocol and during the subsequent visit. This exercise intensity is equivalent to three metabolic equivalents and is the metabolic expenditure associated with healthcare-related activities [2]. During the PPE visit, participants donned PPE after completing the baseline assessments, prior to walking on the treadmill. They doffed PPE after walking on the treadmill, before the rest period. Baseline data and demographics were collected during acclimatization. Core temperature, heart rate, temperature sensation, thermal pleasantness, sweating, thirst, and laboratory temperature and relative humidity were recorded at 5-min intervals throughout the exercise protocol. Mood, grip strength, One-Touch-Stockings (OTS) and Intubation of a Manikin (IoM) tasks, and body weight were assessed at four time points: baseline (before donning PPE), pre-exercise (after donning PPE), post-exercise (after doffing PPE) and post-rest (after rehydration). The time points were chosen to allow assessment of the impact of PPE alone (pre-exercise), PPE and heat stress (post-exercise), and rest and rehydration without PPE (post-rest). Urine specific gravity (USG) was recorded at three time points (baseline, post-exercise, post-rest), because it was not possible to provide a urine sample whilst wearing PPE (pre-exercise).

Core temperature was recorded with an ingestible pill telemetry system (CorTemp, HQ Inc., Palmetto, FL, USA). Participants swallowed the pill 2 h prior to arrival at the laboratory. Heart rate was recorded using an optical heart rate sensor (Polar OH1, Polar Electro, Kempele, FI). Participants rated temperature sensation (perceived body temperature), thermal pleasantness (perceived pleasantness of their thermal environment), sweat and thirst with 100-mm visual analogue scales (VASs) [10–13]. Laboratory temperature and relative humidity were recorded with a data logger (RS-172, RS Components, Corby, UK). Mood was assessed with the Bond–Lader scale, which comprises sixteen 100-mm VAS, that is used to derive the three factors: alertness, contentment and calmness [14]. Grip strength was assessed with a hydraulic hand dynamometer (SH5001, Saehan Corporation, Masan, Korea). Recorded values are the average of three maximum voluntary contractions. The OTS task (CANTABeclipse, Cambridge Cognition, Cambridge, UK) evaluates executive function [15]. During this task participants are shown two displays of three coloured balls in a different order. The aim is to calculate the minimum number of times a ball must be moved to match the two displays. During each assessment participants solved 15 problems. Proportion problems solved correctly on the first attempt (OTS accuracy) and median time to select the correct answer (OTS latency) were recorded. The IoM task is a method of assessing proficiency at endotracheal intubation (Airway Management Trainer, Laerdal Medical, Orpington, UK). The task contains 14 components which must be completed in the correct sequence (Table 1). Each component is awarded a score: 0 = not completed; 1 = partially completed, completed out of sequence or completed after a prompt; and 2 = completed correctly on the first attempt. Total score (out of 28) and time to complete the task were recorded. Additionally, participants rated intubation task effort and performance quality using 100-mm VASs [3]. Body weight was recorded with a digital weighing scale (9207 WH3R, Salter, Tonbridge, UK) and USG with a refractometer (ATC 81150-30, Cole-Parmer, Chicago, IL, USA).

**Table 1.** The components of the Intubation of a Manikin task

1. Place the face mask on the manikin to pre-oxygenate
2. Select the size 7.0 ETT and check the pilot balloon is functional with 20 ml of air (clear syringe)
3. Select the MAC 4 laryngoscope and check that the laryngoscope light is working
4. Give medication with a 20-ml syringe (white syringe)
5. Remove the oxygen
6. Pick up the laryngoscope in the left hand
7. Tilt the head back and insert the laryngoscope into the mouth
8. Obtain a view of the vocal cords without breaking the teeth (only 1 point if teeth are broken)
9. Pick up the ETT with the right hand
10. Successfully insert the ETT into the trachea
11. Inflate the ETT cuff with 20 ml of air (clear syringe)
12. Attach the capnometer, catheter mount and self-inflating bag to the ETT and ventilate the lungs
13. Auscultate both lungs
14. Secure the ETT with a tube tie (half hitch and bow)

ETT, endotracheal tube; MAC, Mackintosh blade. Each component may be awarded 0, 1 or 2 points and thus the maximum achievable score is 28.

Given the exploratory nature of this physiological study, a sample size of 16 was chosen based on previous investigations of thermoregulation and PPE, where 6–18 participants were studied [2–6]. A *post hoc* power calculation was performed to assess the adequacy of the sample using the core temperature data, which demonstrated a power of 0.98 for the interaction effect between PPE and time. Data were analysed with SigmaPlot software (v12.5, Systat, San Jose, CA, USA). Data are presented as mean (standard deviation [SD]), median (interquartile range [IQR]) or *n* (%), as appropriate. Normality and equality of variance were examined with Shapiro–Wilk and Brown–Forsythe tests, respectively. Data were compared with two-way repeated-measures ANOVA. *Post hoc* analysis was conducted with Holm–Sidak methodology. *P*-values are multiplicity adjusted and *P* < 0.05 was considered statistically significant.

## Results

Sixteen healthy participants (eight males) were recruited between February and April 2021. Age 22 (1) years and BMI 22.7 (22.0–24.2) kg·m^−2^. Eight (50%) participants conducted the PPE visit first. The interval between visits was 8 (7–15) days. Laboratory temperature (PPE 21.2 (0.6)°C, control 20.9 (0.7)°C; *P* = NS) and relative humidity (PPE 30 (6)%, control 33 (7)%; *P* = NS) did not differ between visits. Treadmill speed was 3.2 (1.3) km·h^−1^.

**Figure 1.**
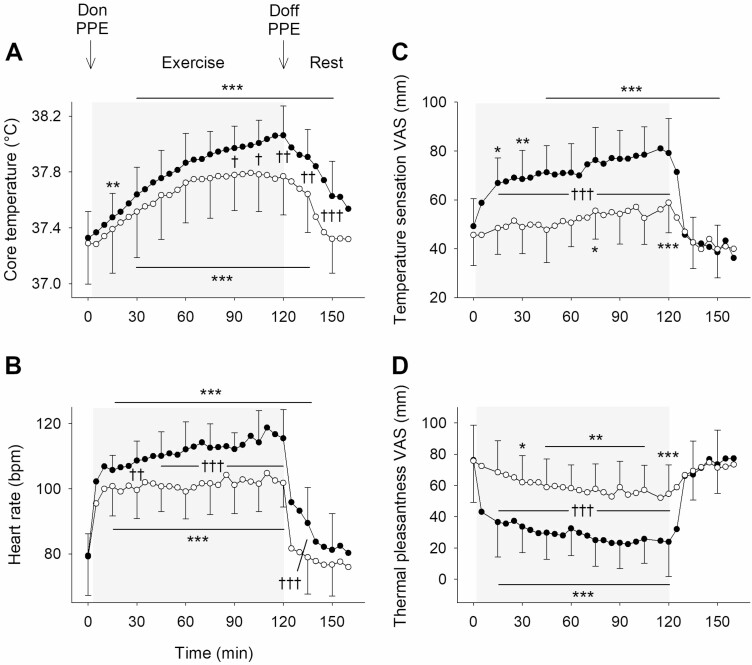
Mean (SD): (A) core temperature; (B) heart rate; (C) temperature sensation VAS; and (D) thermal pleasantness VAS on the PPE visit (black symbols) and the control visit (white symbols). Temperature sensation VAS anchors are: ‘very cold’ (0 mm) and ‘very hot’ (100 mm). Thermal pleasantness VAS anchors are: ‘very unpleasant’ (0 mm) and ‘very pleasant’ (100 mm). Baseline values are displayed at 0 min and the exercise period is denoted by the shaded areas. Difference to the baseline value is indicated by *P < 0.05, **P < 0.01 and ***P < 0.001. Difference between the PPE visit and the control visit is indicated by ^†^*P* < 0.05, ^††^*P* < 0.01 and ^†††^*P* < 0.001.

Core temperature did not differ between visits at baseline (*P* = NS) but was greater on the PPE visit from 90 min of exercise until the end of the rest period (*P* < 0.05; Figure 1A). Maximum core temperature was 38.1 (0.2)°C on the PPE visit and 37.8 (0.3)°C on the control visit. Heart rate did not differ between visits at baseline and at the end of the rest period (*P* = NS), but was greater on the PPE visit from 30 min of exercise until 15 min of rest (*P* < 0.01; Figure 1B). Maximum heart rate was 121 (9) bpm on the PPE visit and 109 (9) bpm on the control visit. At baseline and throughout rest temperature sensation and thermal pleasantness did not differ between visits (*P* = NS; Figure 1C and D), but throughout exercise participants felt hotter and found their thermal environment less pleasant on the PPE visit (*P* < 0.001). Maximum temperature sensation was 86 (10) mm on the PPE visit and 64 (10) mm on the control visit. Minimum thermal pleasantness was 14 (13) mm on the PPE visit and 44 (19) mm on the control visit.

**Figure 2.**
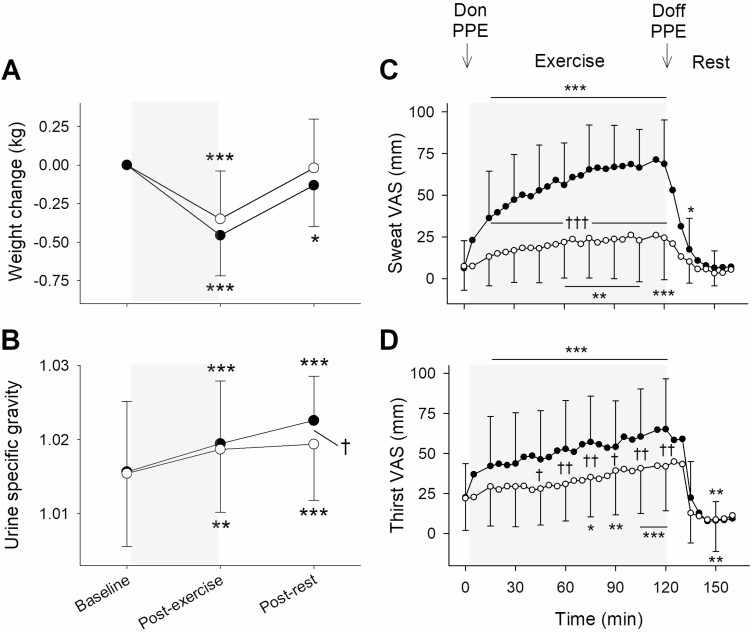
Mean (SD): (A) weight change from baseline; (B) urine specific gravity; (C) sweat VAS; and (D) thirst VAS on the PPE visit (black symbols) and the control visit (white symbols). Sweat VAS anchors are: ‘no sweat’ (0 mm) and ‘soaked and dripping with sweat’ (100 mm). Thirst VAS anchors are: no thirst” (0 mm) and ‘worst possible thirst’ (100 mm). Baseline values are displayed at 0 min and the exercise period is denoted by the shaded areas. Difference to the baseline value is indicated by *P < 0.05, **P < 0.01 and ***P < 0.001. Difference between the PPE visit and the control visit is indicated by ^†^*P* < 0.05, ^††^*P* < 0.01 and ^†††^*P* < 0.001.

Body weight decreased following exercise on both visits (*P* < 0.001; Figure 2A). Post-exercise it decreased by 0.46 (0.26) kg on the PPE visit and by 0.35 (0.31) kg on the control visit. Post-rest body weight was not different to baseline on the control visit (*P* = NS), but it remained less than baseline on the PPE visit (*P* < 0.05). USG was greater than baseline post-exercise and post-rest on both visits (*P* < 0.01), but post-rest it was greater on the PPE visit than on the control visit (*P* < 0.05; Figure 2B). Maximum USG was 1.023 (0.006) on the PPE visit and 1.020 (0.008) on the control visit. Neither sweat VAS nor thirst VAS differed between visits at baseline or during the rest period (*P* = NS; Figure 2C and D). Sweat VAS was greater throughout the exercise period on the PPE visit than on the control visit (*P* < 0.001) and thirst VAS was greater on the PPE visit from 45 min until the end of the exercise period (*P* < 0.05). Maximum sweat VAS was 77 (25) mm on the PPE visit and 32 (27) mm on the control visit. Maximum thirst VAS was 73 (28) mm on the PPE visit and 49 (27) mm on the control visit.

**Figure 3.**
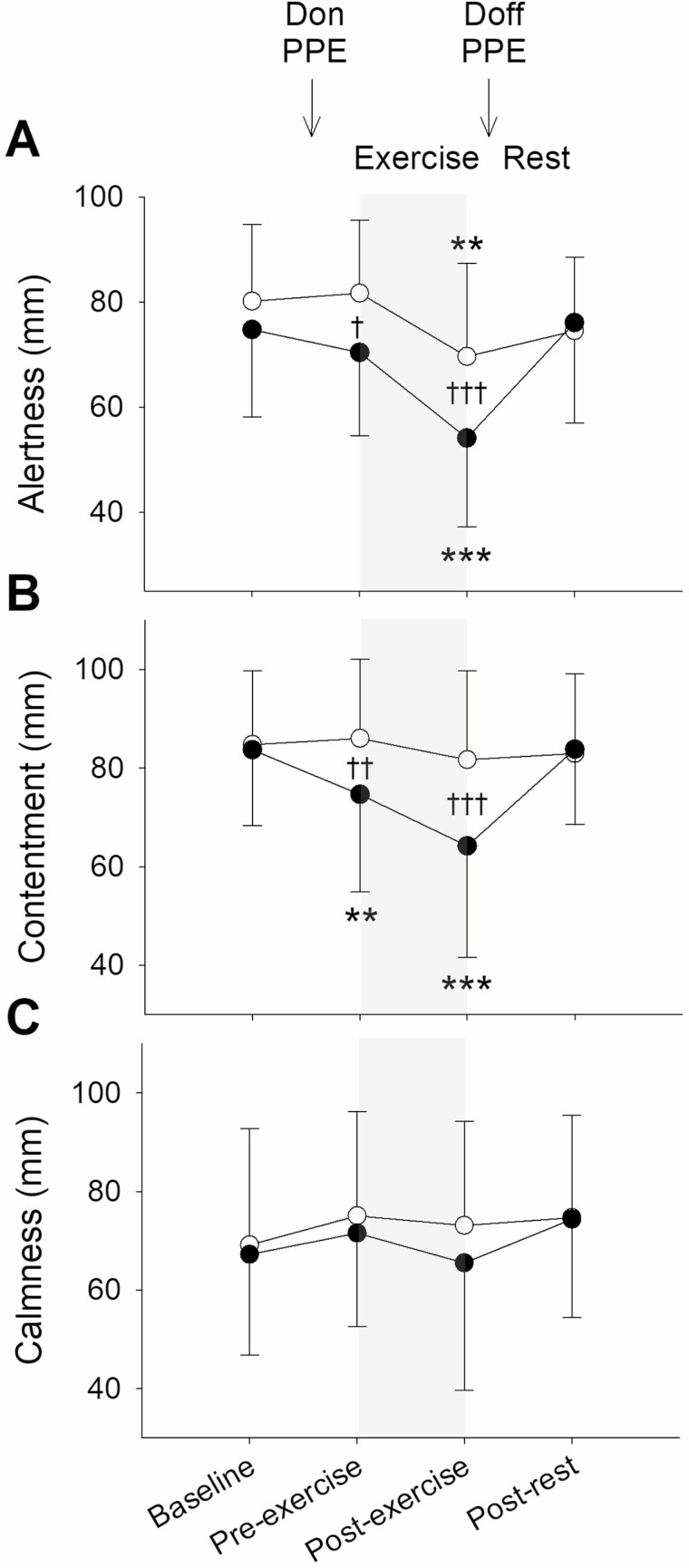
Mean (SD): (A) alertness VAS; (B) contentment VAS; and (C) calmness VAS on the PPE visit (black symbols) and the control visit (white symbols). The exercise period is denoted by the shaded areas. Difference to the baseline value is indicated by **P < 0.01 and ***P < 0.001. Difference between the PPE visit and the control visit is indicated by ^†^*P* < 0.05, ^††^*P* < 0.01 and ^†††^*P* < 0.001.

**Figure 4.**
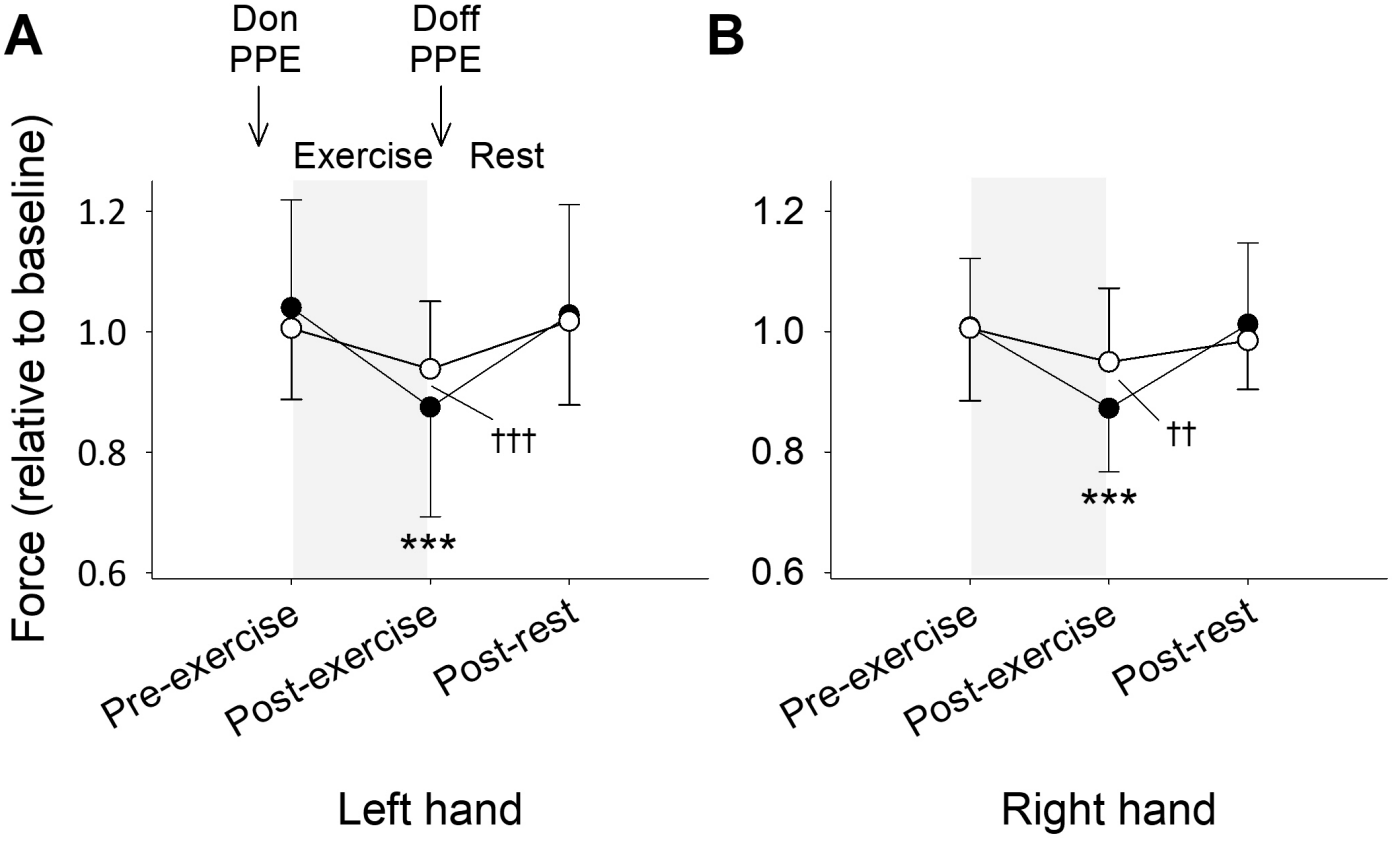
Mean (SD): (A) left; and (B) right-hand grip strength (relative to baseline) on the PPE visit (black symbols) and the control visit (white symbols). The exercise period is denoted by the shaded areas. Difference to the baseline value is indicated by ***P < 0.001 and difference between the PPE visit and the control visit is indicated by ^††^*P* < 0.01 and ^†††^*P* < 0.001.

**Figure 5.**
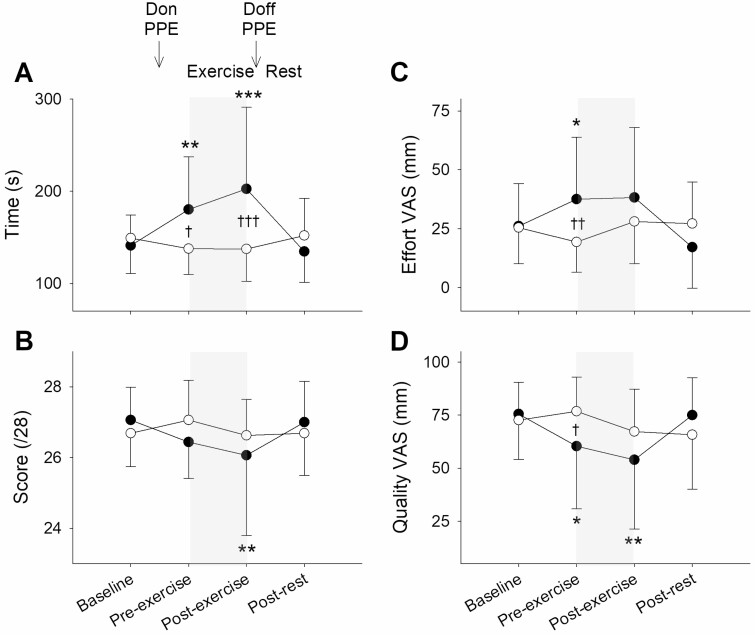
Mean (SD): (A) intubation task time; (B) intubation task score; (C) intubation task perceived effort VAS; and (D) intubation task perceived quality VAS on the PPE visit (black symbols) and the control visit (white symbols). Effort VAS anchors are: ‘very easy’ (0 mm) and ‘very difficult’ (100 mm). Quality VAS anchors are: ‘very bad’ (0 mm) and ‘very good’ (100 mm). The exercise period is denoted by the shaded areas. Difference to the baseline value is indicated by *P < 0.05, **P < 0.01 and ***P < 0.001. Difference between the PPE visit and the control visit is indicated by ^†^*P* < 0.05, ^††^*P* < 0.01 and ^†††^*P* < 0.001.

Participants felt less alert and less content pre- and post-exercise on the PPE visit than on the control visit (*P* < 0.01), but calmness did not differ between the visits (*P* = NS; Figure 3). Minimum alertness was 53 (17) mm on the PPE visit and 67 (16) mm on the control visit. Minimum contentment was 63 (22) mm on the PPE visit and 77 (18) mm on the control visit. Minimum calmness was 57 (22) mm on the PPE visit and 62 (23) mm on the control visit. Grip strength was less than baseline in the right (−41 (51) N; *P* < 0.001) and left (−41 (66) N; *P* < 0.001) hands post-exercise on the PPE visit, but no changes were observed on the control visit (*P* = NS; Figure 4). OTS accuracy and OTS latency did not differ at any of the time points and neither parameter differed between the visits (*P* = NS). At baseline and post-rest, intubation time did not differ between the visits (*P* = NS), but pre-exercise (+43 (66) s; *P* < 0.05) and post-exercise (+65 (103) s; *P* < 0.01) it was greater on the PPE visit (Figure 5). Intubation score was less than baseline post-exercise on the PPE visit (−1 (2); *P* < 0.01) but did not differ between the time points on the control visit (*P* = NS). Perceived intubation effort was greater (+18 (22) mm; *P* < 0.01) and perceived quality was lower (−16 (29) mm; *P* < 0.05) on the PPE visit than on the control visit pre-exercise, but neither parameter differed between the visits at baseline, post-exercise or post-rest (*P* = NS).

## Discussion

This study quantified the heat stress associated with wearing aerosol PPE in a simulated hospital environment and examined its impact on mood, cognitive and motor function, and task performance. Wearing aerosol PPE resulted in heat exhaustion and negatively impacted mood, grip strength and task performance.

The changes in core temperature demonstrate that wearing aerosol PPE is a heat stress. The increase followed by plateau in core temperature during the control visit is an expected consequence of exercise and is caused by a transient heat imbalance [16]. By contrast, the constantly rising core temperature during the PPE visit is the pattern observed when heat production exceeds heat loss [17]. This is characteristic of heat stress and is observed in other scenarios [2,3,17,18]. During heat stress heart rate increases because vasodilation induces a baroreceptor-mediated increase in cardiac output [19]. Therefore, the increased heart rate during the PPE visit is an expected consequence of the rise in body temperature. Likewise, the greater weight loss and the greater increase in USG on the PPE visit occur because the greater core temperature increases sweating [20].

Both PPE and heat stress negatively impact mood, motor and cognitive function, and task performance. It is important, therefore, to separate the effects of wearing aerosol PPE, which occur immediately after donning PPE (pre-exercise), from the effects of the heat stress, which occur after wearing PPE for a period of time (post-exercise) [3,4,21]. Grip strength was not affected by donning PPE but decreased post-exercise. This was anticipated because hyperthermia decreases neural drive to skeletal muscle and reduces maximal force production, but wearing non-medical PPE does not [4,22]. By contrast, intubation task performance decreased after donning PPE and then decreased further after exercise. The intubation task has not previously been used to evaluate performance in PPE; however, conceptually it tests fine motor skills and short-term memory. Manual dexterity is impaired by other forms of PPE and short-term memory is impaired by hyperthermia and so the present results align with previous work [4,21]. Subjectively, healthcare workers report that medical PPE adversely affects fine motor skills and short-term memory [7,23]. However, although participants reported a drop in performance after donning PPE, they did not notice the further decrement following exercise. This lack of awareness has been observed previously during heat stress and has patient safety implications [24]. Medical PPE and heat stress have a negative effect on mood and the reduction in alertness and contentment concur with these previous findings [7]. The lack of change in calmness was unexpected but may be due to the positive effect of exercise [25]. Contrary to the hypothesis, executive function was unaffected by wearing aerosol PPE or the associated heat stress. This unexpected result is likely because the severity of the heat stress was insufficient. It is not known if wearing PPE affects executive function, but heat stress only has a negative impact when core temperature increases 1.0°C or more, a rise not seen in this study [5,26].

Collectively, the results demonstrate that wearing aerosol PPE for 2 h results in heat exhaustion (core temperature >38°C, heart rate >100 bpm, excessive sweating and thirst, impaired short-term memory and motor function) [27]. Because the insulative properties of aerosol PPE are independent of body temperature, it can be extrapolated that, if exercise had continued beyond 2 h, core temperature, heart rate and dehydration would have continued to increase [28]. It is likely that mood, motor function and task performance would also have deteriorated further. Ultimately, if exercise was not terminated and PPE doffed, heat exhaustion would have progressed to heat stroke (core temperature >40°C, encephalopathy, multi-organ dysfunction), with potentially life-threatening consequences [27]. Conversely, the post-rest results demonstrate that 30-min breaks alleviate the heat stress and its associated performance decrements. However, the continued dehydration at the end of the rest period demonstrates that 500 ml of electrolyte rehydration drink every 2 h is insufficient. The long-term impact of aerosol PPE was not specifically examined in this study. However, the results support the hypothesis that repeated exposure increases the risk of fatigue, burnout and long-term physical and mental illness [8,9].

This study has potential implications for healthcare workers, healthcare organizations and PPE manufacturers. The use of aerosol PPE is likely to remain commonplace for the foreseeable future. Whilst the use of aerosol PPE is essential in many circumstances, this study demonstrates objectively that its use has side-effects which could impact negatively on healthcare workers’ well-being and performance. As many of these negative effects are time-dependent and reversible, healthcare organizations should consider limiting the time healthcare workers spend wearing aerosol PPE without breaks. Additionally, as participants are not always aware of performance decrements, this study supports the usage of ‘buddy’ systems which enable healthcare workers to check on each other when wearing aerosol PPE [29]. PPE manufactures should develop heat-permeable garments which would allow healthcare workers to wear aerosol PPE for longer without experiencing the negative side-effects. However, before implementing any large-scale interventions, it is recommended that the results of this study are verified by larger studies conducted in real-world hospital environments.

A limitation of this study is its generalizability. The study was conducted in a human performance laboratory and the participants were neither healthcare workers nor heat acclimatized. Whilst it is impossible to recreate in a laboratory the emotional strain of caring for critically ill patients, it is possible to recreate the thermal conditions [2]. Therefore, the changes in core temperature, heart rate and hydration are generalizable to the hospital environment. The negative impact of PPE and heat stress on mood and grip strength has been reported in a range of populations and so these findings are likely also generalizable [4,7]. As this is the first time the intubation task has been used to evaluate the impact of either PPE or heat stress, the generalizability of this result is less certain. However, given that fine motor skills and short-term memory are both adversely affected by a variety of forms of PPE and heat stress, it is plausible that healthcare workers would experience a similar performance decrement [4,7,21,23]. Furthermore, older and less healthy individuals are more susceptible to the consequences of heat stress and therefore vulnerable healthcare workers might experience the adverse effects of aerosol PPE to a greater degree than the young healthy participants in the present study [26,30].

In conclusion, this study is the first to quantify the heat stress associated with aerosol PPE usage and to objectively evaluate its impact on mood, motor and cognitive function, and task performance. The results demonstrate that wearing aerosol PPE results in heat exhaustion and negatively impacts mood, motor function and task performance. Aerosol PPE usage is likely to remain prevalent in the future; therefore, strategies should be developed to mitigate its impact.

## Supplementary Material

kqac114_suppl_Supplementary_Material
