# Supplementary material for: Heat stress associated with aerosol PPE and its impact
Source: Occup Med (Lond). 2022 Oct 25;73(3):120–7. doi: 10.1093/occmed/kqac114 (PMC10132207; doi:10.1093/occmed/kqac114)
Supplement: kqac114_suppl_Supplementary_Material [file kqac114_suppl_supplementary_material.docx]

**Supplementary figure 1** A subject wearing (A) aerosol personal protective equipment (PPE); and (B) standard surgical attire.


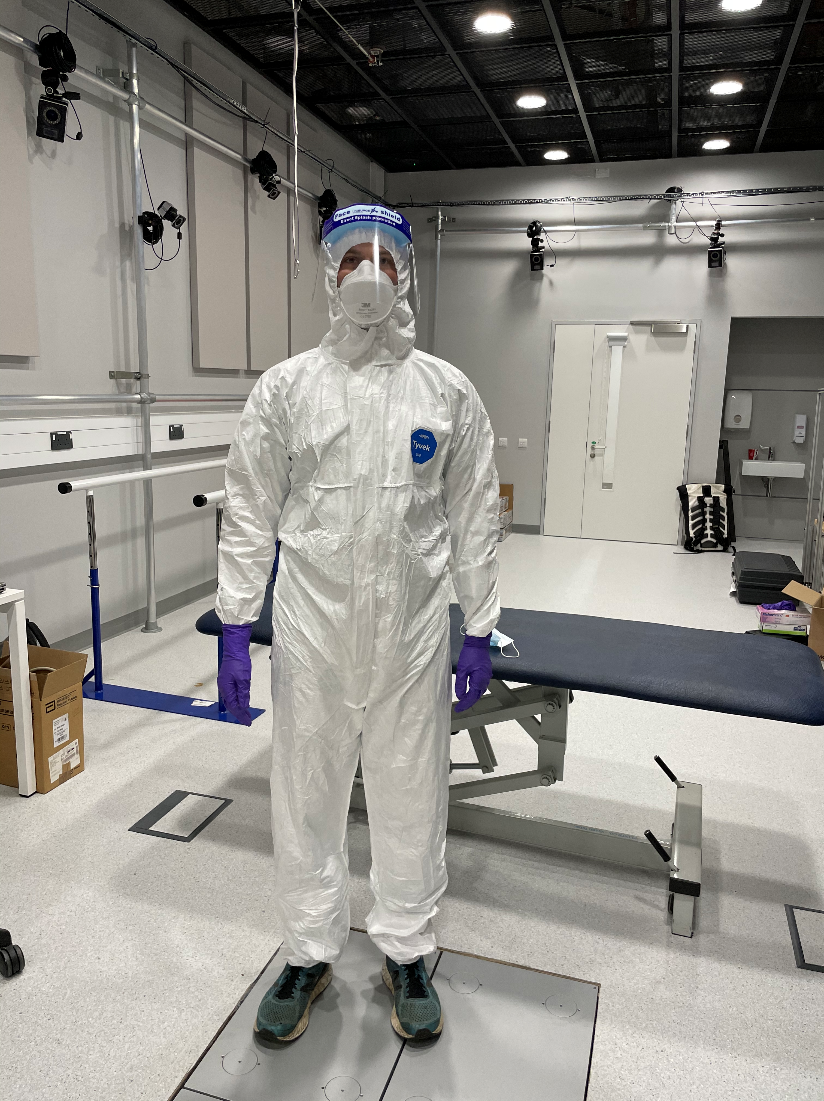

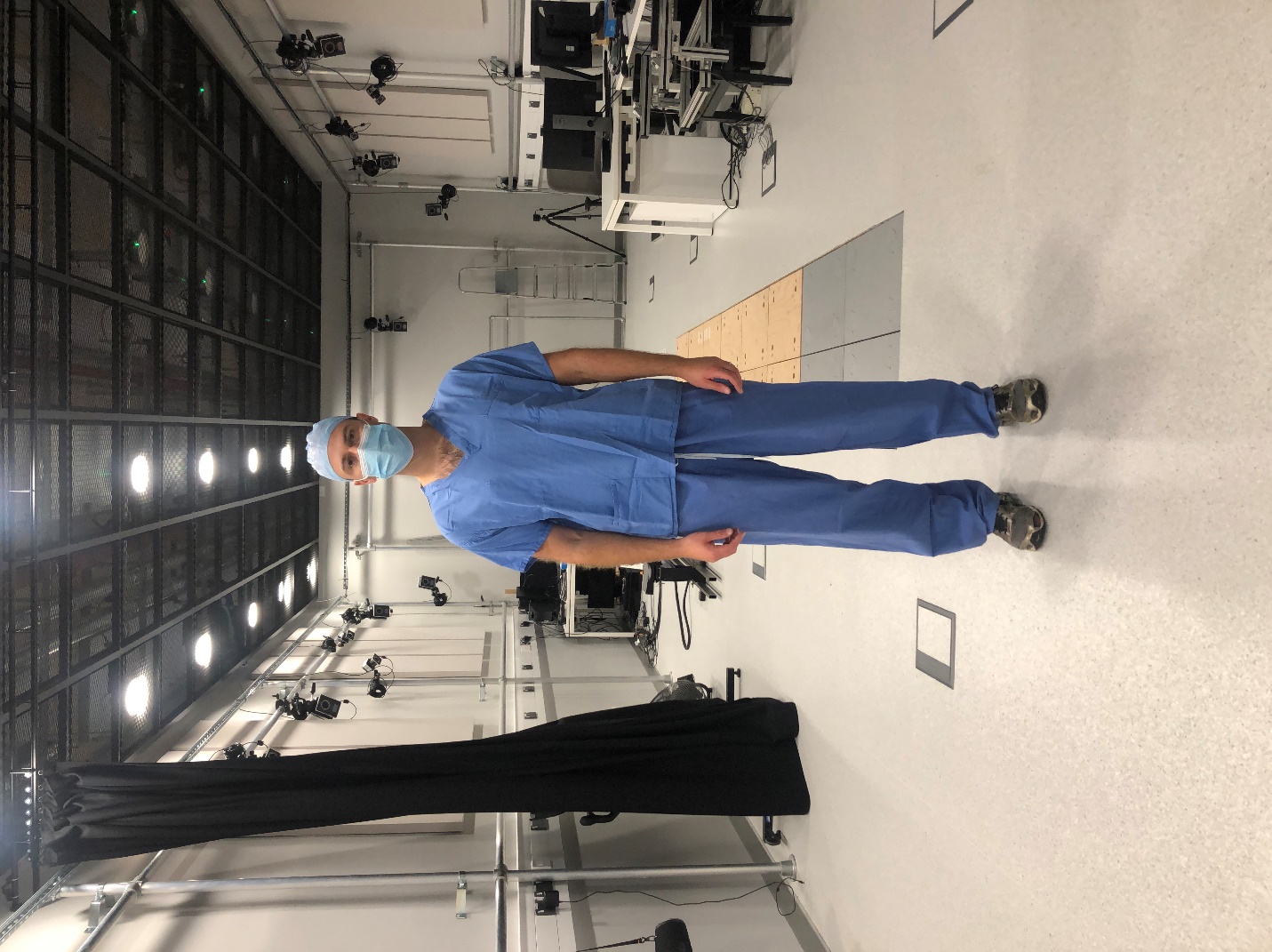


B

A
